# Striving to reduce suffering: A Phenomenological Study of nurses experience in caring for children with cancer in Ghana

**DOI:** 10.1002/nop2.650

**Published:** 2020-10-06

**Authors:** Ruth Nimota Nukpezah, Fatemeh Fomani Khoshnavay, Marzieh Hasanpour, Alireza Nikbakht Nasrabadi

**Affiliations:** ^1^ Department of Pediatric Nursing School of Nursing and Midwifery International campus‐Tehran University of Medical Sciences Tehran Iran; ^2^ Department of Pediatric Nursing School of Nursing and Midwifery Tehran University of Medical Sciences Tehran Iran; ^3^ Department of Pediatric Nursing NIDCAP Professional Spiritual Health Branch of Rresearch Center of the Quran; Hadith and Medicine School of Nursing and Midwifery Tehran University of Medical Sciences Tehran Iran; ^4^ Department of Medical and Surgical Nursing School of Nursing and Midwifery Tehran University of Medical Sciences Tehran Iran

**Keywords:** cancer, caring, children, experience, Ghana, hermeneutic, nurses, phenomenology, suffering

## Abstract

**Aim:**

To provide insights into nurses lived experiences in caring for children with cancer.

**Background:**

Little is known about the paediatric oncology nurses shared practices of caring for children with cancer in Ghana.

**Design:**

A hermeneutic phenomenological qualitative study.

**Methods:**

A semi‐structured interview with 14 purposely sampled Ghanaian paediatric oncology nurses. Findings were analysed using Diekelman, Allen and Tanner's approach.

**Results:**

The theme "Striving to reduce suffering" and three relational subthemes: "knowing children's needs," “Rendering a hopeful fight” and “Ensuring continuity and coordination of care” emerged. Increased awareness of this phenomenon for the nurses who care for these children is vital to ensure quality and holistic care that is meaningful and satisfying by nurses for children with cancer. Paediatric oncology nurses can use the result of the study to evaluate their caring practice and as an avenue to develop better caring practice.

## 1 INTRODUCTION

Nurses play an indispensable role in caring for children with cancers. Paediatric oncology nurses not only provide technical and scientific knowledge, but also give humanized care to promote the health, quality of life, comfort and well‐being of the sick child (Al Balushi, 2019; Suryani et al., 2018). Other studies mentioned that the oncology nurses concern for a child with cancer is shown through nursing creativity to find ways to communicate and give tender loving care to the child (Montgomery et al., 2017; Nafratilova et al., 2018). Paediatric oncology nurses must understand the tasks involved in their nursing duties to improve the quality of life of patients (Suryani et al., 2018). From the literature reviewed, there were paucities of studies on the experiences of nurses who care for children with cancer. This study will provide the Paediatric oncology nurses (Participants) with the means to tell their stories about their shared patterns of behaviours, feelings and actions that they use in caring for children with cancers. Once these experiences, feelings and behaviours are identified, it can provide a more comprehensive understanding on the concept of care and provide a foundation for important nursing education and formulation of nursing theory to strengthen patient care. Therefore, this study aims to understand the life world of paediatric oncology nurses in Ghana.

### 1.1 Background

Nursing is a caring profession. When providing care to children with cancer, the nurses caring work is more prominent. Globally, approximately 300,000 children under age 20 are diagnosed with cancer each year (Slone et al., 2018), and the number of deaths caused by cancer in children aged 3–15 is more than any other disease (Gouda et al., 2019).

A study in Ghana reported that there are more than 250 cases of childhood cancer each year in Ghana (Renner et al., 2018). This situation places several demands on nurses who must take care of not only the children but their families as well. Among health professionals caring for children with cancer, nurses stand out. They play a supporting role and as well as deal directly with children every day (Nukpezah et al., ). Nurses are committed to participating in the human experience of living with illnesses. Underlying this commitment is a commonly held value of developing a meaningful caregiver relationship as a way to know and understand patients experience.

Moreover, caring has strong relationships with cultural and social characteristics (Compton et al., 2019). Recent care approaches in paediatric oncology nursing showed that nurses experiences, family‐centred care, technology‐based care, primary care of a child, survivor care, home care and non‐pharmacological care (Toruner & Altay, 2018). Some nurses experience giving “futile care,” a situation where the care given to a client does not contribute to improving the physiological status of the client (Bahramnezhad et al., 2014).

Besides, oncology nurses experience many aspects of nursing care that are intangible. The intangible skills described by nurses include communication skills, the courage to be with patients in their suffering, the ability to recognize their limitations, ability to seek help and learn from their mistakes (McKenna, 2018). Without enough attention to these difficult‐to‐measure skills, these skills will be under‐estimated in the growing technology‐centric healthcare system.

The lived experience of the paediatric oncology nurses depends to a great extent on; their immediate sociocultural context, quality of care and an accessible healthcare system (Al Zoubi et al., 2020; Dilek Konukbay et al., 2019).

Indeed, there is a growing body of literature addressing the experiences of the adult oncology patients and their families (Al Zoubi et al., 2020; Compton et al., 2019; Handberg et al., 2018; Tutelman et al., 2019; Witte & Handberg, 2019). However, literature exploring the caring experiences of paediatric oncology nurses is scarce. For nurses who provide care to children with cancer globally and Ghana, there is a gap for qualitative studies to be completed to fully grasp and gain a deep understanding of the lived experience.

Thus, the review of the literature supports the notion that it is timely and appropriate to explore nurses' experiences of caring for hospitalized children with cancer and their families in other to understand the nurses better. Learning about the insights, knowledge, skills and attitudes of nurses will help to supply expert knowledge and help to validate their personal experiences (Montgomery et al., 2017). Increased awareness of this phenomenon is vital to ensure quality and holistic care that is meaningful and satisfying by the nurses for patients. Thus, this study explored the lived experience of nurses caring for children with cancer in Ghana by the use of the phenomenological method of enquiry.

### 1.2 Aim

To describe nurses experiences in caring for children with cancer.

### 1.3 Research question

The central research question that instructed this research study was as follows:

“What is the lived experience of nurses caring for children with cancer in Tamale, Ghana?”.

### 1.4 Probing questions

Can you explain it more? What were your feelings at that moment? What did you think about the situation? What is the meaning of caring for children with cancer?

## 2 METHODS

### 2.1 Study design

The study employed a hermeneutic phenomenological qualitative study. A hermeneutic phenomenological method elicits the perspective of participants on the meaning of “being in the world” (Heidegger, 2010). Hermeneutic phenomenology, as described by Hans Georg Gadamer philosophies, was adopted (Ramsbotham, 2019). The concept of, hermeneutic circle of understanding, prejudice, the rhetoric of understanding, historicity, the fusion of horizons and lived experience from Gadamer was embraced throughout the conduct of this study. The hermeneutic method was used to uncover meanings embedded in nurses narratives about caring for children diagnosed with cancer. The meaning of these experiences lies in the interpretation of the experience from the individual nurse unique perception (Ramsbotham, 2019).

### 2.2 Inclusion criteria

The following were the criteria set for inclusion:

- Registered Nurses (Male and female) at the paediatric oncology ward of Tamale Teaching Hospital (TTH) Ghana.
- Nurses with at least two years of experience in Paediatric cancer care.
- Nurses who are willing to participate and talk about their experiences of caring for children with cancer.

It was expected that the nurses are familiar with the practical environment and have a meaningful experience that can be used for reference.

### 2.3 Data collection method

Individual, in‐depth, semi‐structured interviews that were conversational in style was used for this study. Before interviewing participants, the nurses at the Paediatric Cancer Unit were duly informed about the purpose and procedure involved in the study. For those willing to participate, interviews were scheduled at their convenience in the nurse's private room. Participants were asked the question: “Please describe your experiences in providing nursing care to children with cancer?”. This question was further followed with probing questions such as: Can you explain it more? What were your feelings at that moment? What did you think about the situation? What is the meaning of caring for children with cancer? The interviews ranged from 45 min–60 min. Throughout the interviews and after each question or comment, the participants were given enough time to tell their stories. Sampling was stopped when no other new findings emerged following the interview with the 14 purposely chosen nurses (data saturation). All conversations were recorded using a digital voice recorder (Dictaphone) with permission of the participants. The recorded data were transcribed verbatim immediately after the end of each interview and saved as computer files. Participants were named, P1–P14 to ensure anonymity.

### 2.4 Data analysis

Searching for a method congruent with Gadamer's philosophy, we adapted the Diekelmann, Allen and Tanner's 7 step analysis approach (Diekelmann et al., 1989). The Principal investigator [PI] collected and transcribed the interviews according to the first stage of Diekelman analysis approach. The interview content was read several times to fully understand the participants' accounts of their lived experience in caring for children with cancer, and an interpretive summary was written during the second stage to comprehend the implicit meanings. This step required much patience, as the researcher was expected to remain open, embrace more universal points of view and constantly move from the whole to the part and back to the whole again [fusion of horizons] (Ramsbotham, 2019). Accordingly, meaning units were extracted in a stepwise manner, following the hermeneutic circle of immersion in the participants’ textual transcribed narrative accounts. In the third stage, subthemes and a theme were extracted from the meaning units and appropriate Phrases were constructed for each of them. At the fourth stage, all members of the research team discussed the transcribed interviews via emails and chat. All disagreements on interpretations were discussed, and a group consensus was reached. In the fifth stage, by comparing and contrasting the texts, common meanings were further distinguished and explained using the hermeneutic circle. After comparing the theme and the subthemes in the sixth stage, the relationship between them was determined. At the seventh stage, through discussions and further analysis of all data, the themes cutting across all narratives were further explored, discrepancies were discussed, common agreements made and imputed in the final draft (Diekelmann et al., 1989). Data collection and analysis were performed from August 2019–April 2020.

### 2.5 The trustworthiness of the study

To ensure trustworthiness, the concepts of credibility, confirmability, dependability and transferability were carefully followed (Lincoln & Guba, 1986). We maintained credibility and confirmability of the study through close interaction with participants, prolonged and continuous engagement with the data and the implementation of a team approach throughout the stages of the study. The interviews were transcribed by the PI instantly after the end of each interview. The analysis of the data was carried out in a stepwise manner, following the hermeneutic circle. All evidence and documents have been kept safely to maintain dependability (repeatability). Also, the findings from the participants were presented using thick descriptions, the context is described in sufficient detail and quotes from the participants were used to allow the reader to make a transferability judgment (Lincoln & Guba, 1986).

### 2.6 Ethical issues

This study had approval from the Research Ethics Board of School of Nursing and Midwifery & Rehabilitation, Tehran University of Medical Sciences Tehran, Iran, on 11 July 2019 (approval code: IR. TUMS.VCR.REC.1398.273) and in Ghana by the Korlebu Teaching Hospital ethical review board on 31 December 2019 (approval code: KBTH IRB/000127/2019). The three ethical principles of the Belmont Report were taken into account (respect of a person, beneficence and justice), by clearly explaining the aims of the study, maintaining their anonymity in transcription by naming them, P1‐P14 and preserving their right to withdraw from the study. Participants were well informed about the aim of the study and were requested to sign an informed consent form.

## 3 RESULTS

### 3.1 Demographic representation of participant

The sample size was made of a diverse group of state registered nurses which represents the demographic make‐up of the region where this study took place and all had a detailed description of their experiences in cancer care. Demographic data are shown below in Table 1.

**Table 1:** Demographics of participants

Participants	Gender	Age	Education	Years of Experience	Interview Duration	No of Interviews
1	Male	29	B.Sc. Degree	2	45 min	2
2	Male	34	B.Sc. Degree	6	47 min	1
3	Male	36	B.Sc. Degree	4	1 hr	2
4	Male	40	B.Sc. Degree	12	50 min	1
5	Female	40	B.Sc. Degree	11	48 min	2
6	Male	35	B.Sc. Degree	7	55 mins	1
7	Female	36	Masters	12	49 mins	1
8	Female	45	B.Sc. Degree	9	58 min	1
9	Male	28	B.Sc. Degree	2	45 min	1
10	Male	36	B.Sc. Degree	6	46 min	1
11	Male	36	B.Sc. Degree	6	1 hr	1
12	Female	34	Masters	11	59 min	1
13	Female	36	Masters	11	46 min	1
14	Male	34	B.Sc. Degree	9	49 min	1

Note

Authors construct (2020).

Following an in‐depth analysis of the transcripts one theme; striving to reduce suffering and three related subthemes of meaning: “knowing children's needs,” “Rendering a hopeful fight” and “Ensuring continuity and coordination of care” were identified that explicated the participants lived experience as shown in Table 2. The extracts chosen here are those considered to represent those most useful to practice and future research.

**Table 2:** Extraction of the main theme and subthemes from the meaning units

Meaning units	Subthemes	Theme
Involving children in play activities. Constantly observing children.	Knowing children's needs	
Offering education to children and families. Offering curative, palliative and/or end of life care.	Rendering a Hopeful fight	Striving to reduce suffering.
Encourage families to complete children's treatment cycles. Acting as liaison between patients and other oncology team members.	Ensuring continuity and coordination of care	

Note

Authors construct (2020).

### 3.2 Striving to reduce suffering

Interviews with the participants showed that nurses, through teamwork approach with other healthcare personnel's and involvement of patients and families, rendered diverse forms of care to reduce the oncology children's pain and sufferings. The care they gave included the relief of suffering, which was done through the assessment of children's pain; the treatment of pain (by the use of prescribed medication and other nursing care approaches, such as diversional therapy); and the management of identified physical; psychosocial; and spiritual problems. The care nurses gave to these children and their families often start at the time of diagnosis and continued throughout life and beyond. Nurses attend to aspects of care that ease the sufferings of patients and their families. When the diagnosis is beyond curative treatment, they help in promoting a peaceful death. They described aspects that relieved sufferings for the patient as well as the patient's families.

#### 3.2.1 Knowing children's needs

Concerning the notion of knowing paediatric oncology patient's needs, the nurses' stories showed that an important aspect that affects the behaviour of nurses in caring for children diagnosed with cancer is the understanding of the patient. Nurses routinely conduct a comprehensive health history and physical assessment. The findings that nurses get from these daily findings help them to gradually understand the trajectory of the disease and events that may occur in advance to the patient. These experiences helped to build a therapeutic relationship between the paediatric oncology nurses, the oncology patient and the patient's family.

The following quotation illustrates one of the participants’ expression as one them said:

Observing the kids and asking the kids preferences as I observe them, their hobbies and then their interests such as reading a novel e.t.c. And asking them about whether they are bored? And also, about their wants and needs, helps me to know my patients and understand them. [P1]

Other participants also mentioned:

I interact and play with the children and sometimes I even get some problems from the child which the parents don't know about. [P6]

The relationship between me and the child and their family are always cordial, you have to be friendly with the parents because they do give us more information about their children. [P8]

#### 3.2.2 Rendering a hopeful fight

Participants talked about how they cared for patients with cancer by instilling the hope of recovery in them. Participants mentioned that managing children with cancer means helping patients face an uncertain future. The nurses described the importance of maintaining hope for remission or cure when cancer patients are receiving treatment and life prospects are uncertain. The nurse talked about the importance of maintaining the vitality of hope when the patient enters the final stage of life or when the patients are showing signs of daily improvements in their condition. Participants revealed that they gave curative and palliative care (PC) to most children. For other children, end of life (EOL) care was given if or when disease‐modifying efforts fail or become unsuitable. In rendering a fight against cancer and, most nurses offered clients and their family education about the condition. They provided the families chemotherapy medications through the use of palliative care approaches and also encouraged the patients and their families.

In the following quotations, participants described the therapeutic interventions that they offered to patients and their families:

We educate them every day when we go by their beds to give them their medications, which are mostly chemotherapy drug, we ask them if there is anything they need to know. If they are anxious, they don't know we consistently kind of explain things to them and then cheerfully tell them that their children will be fine [P5]

We try to be there and try to support the family as much as we can, we try to make sure the children comfort needs are met, by given them their prescribed medications and other life support treatments [P6]

I became very close to the child and I will forever remember that child and his parents after he died. The parents needed a lot of support and we had to comfort them. [P3]

#### 3.2.3 Ensuring continuity and coordination of care

The continuity and coordination of care were recognized as very significant by the participants as they told stories about how they used various strategies which include constant and continuous conversations with the parents of the children about the importance and multiple ways to ensure compliance with treatment. The participants explained their roles of harmonizing holistic and total care as they form a link between the family and the other oncology team members.

The participants stated that:

We inform the other oncology team members about the progress the child has made, because we stay more with the patient and we are the best people to identify either positive and negative changes in their condition and we work to communicate this changes to the rest of the oncology team members. [P7]

We encourage families to continue to make sure that they complete their treatment cycles even when their children begin to show signs of getting better, this is because most families start treatment but find it very difficult to complete because it takes their finances and time from them. Thus, to ensure that all our previous supportive care is not in vain, we constantly motivate them by giving them words of encouragements. [P12]

……To reduce abandonment rates, we provide counselling to empower families with the information on not only the progress of the treatment but to also update them of what to expect in terms of its intensity of time, money and every other thing involved. We do all this so that there will be treatment compliance so that they complete their chemotherapy cycle [P11]

## 4 DISCUSSION

The purpose of this study was to understand the caring practice of paediatric oncology nurses in Ghana. The participants' stories unlocked a wealth of past experiences that shaped and moulded their present world and developed “thick” descriptions that illuminated how they had come to interpret and give meaning to their day‐to‐day experiences of caring for children with cancer. The stories told by the nurses remained with them and according to Gadamer, these experiences formed the historical reality of their being as well as their horizon of understanding (Ramsbotham, 2019).

The participants acknowledged that to care for children with cancer, they had to “strive to reduce suffering”. In this study, the nurses strived to reduce suffering, by “Knowing the children's needs,” “rendering a Hopeful fight” and involved themselves in “Ensuring continuity and coordination of care.”

Striving to reduce suffering was well illustrated by the countless efforts undertaken by nurses to manage the pain and anxiety experienced by children with cancer. Alleviation of Suffering is the main essential category of all caring acts. This is because, suffering gives caring its character and identity and all forms of caring to aim in one way or another to alleviate suffering (Suryani et al., 2018). If the process to reduce suffering is met with challenges, it can lead to futile care (Bahramnezhad et al., 2014). In this present study, the paediatric oncology nurses helped to reduce patients’ sufferings, because they were in constant touch with the clients compared with other oncology team members and hence promoted a hopeful fight against cancer and they worked tirelessly to ensure continuity and coordination of care for the patients and their families.

Knowing the child's need refers to the therapeutic relationship between the paediatric oncology nurses, the oncology patient and the patient's family members. Following this, it helped to understand an event as it had meaning for the patient and it helped deepen the understanding of the patient as a unique individual. This type of care is supportive, personalized, comforting and healing (Brimble et al., 2019; Mojen et al., 2018; Wheeler, 2016; Organization, 2014). Other studies have reported by knowing the patient with a chronic health problem such as cancer, nurses were able to observe and assist in the treatment of basic physical assessment findings. These assessment findings include: fatigue, hair loss, depression, nausea, confusion, pain, anger, bleeding, diarrhoea and mouth sores/mucositis. Other findings were insomnia, neuropathy, neutropenia night sweats, weight loss and sensory changes that are found in patients who are on chemotherapy treatment (Linder & Wawrzynski, 2018; Silva et al., 2016; Ward et al., 2020). These participants' stories revealed that they played with the children and regularly watched the children to monitor their progress. They triangulated multiple sources of evidence to formulate assessments results. The nurses considered the child's age, cognitive development level and communication skills. Nurses adoption of pain‐relieving measures depended on their experiences in dealing with pain and their convictions about pain. Nurses are expected to assess children using developmentally appropriate strategies (Enskär et al., 2020). With these competencies at hand, nurses will be able to develop a deeper understanding of the patient as a unique individual.

Participants mentioned that they provided a hopeful fight, by giving holistic care which included giving education on cancer to clients and their family members. The paediatric oncology nurses provided curative and palliative care (PC) for most children. They also stated that for other children, end‐of‐life (EOL) care was given if or when disease mitigation measures fail or become inappropriate. Participants further mentioned that children with cancers and their families were hopeful, relieved and calm when they communicated assessment findings with them. Nurses provide hopeful fight to address patients’ sufferings through effective pain symptom management; they give psychological, social and spiritual support (Al Balushi, 2019). When managing symptoms, nurses administer chemotherapy mediations and also monitors the side effects of such treatments on the patients. Also, nurses conducted an independent intervention to lessen the child's pain by using distraction techniques. They did this by playing games, drawing or walking around with the children. Nurses involve parents in caring for their children through the family‐centred‐care approach of inclusiveness, collaboration and attention to their belief system (Fukumori et al., 2020; Hopia et al., 2019). The nurse's experience will always have to occur in the context of the relationship and interaction with the patient (Borhani et al., 2013). Anecdotal evidence shows that, when nurses show humour in their behaviour, truthfully explanations, being caring and carrying out activities together with the patient and give skilled care, it positively increases cancer patients hope of living a meaning life. A trustworthy, friendly, enthusiastic, honest and affectionate paediatric nurse is essential for building a battle of hope against cancer (Offen, 2015). However, another study reported that health professionals could not communicate with cancer patients due to lack of appropriate communication skills or due to a lack of confidence in responding to emotional issues (Tafjord, 2020), there is complexity in providing information to relatives of a sick child (Gee et al., 2020). However, in this current study, the nurses gave education, information and support to clients.

Furthermore, paediatric oncology nurses also work to “ensuring continuity and coordination of care”. They did this by constantly reminding the children and their families about the importance of follow‐up treatment for the multiple cycles involved in chemotherapy needed by the child. The nurses in this study sometimes bought gifts for the children. They liaised with financial support groups such as “world child cancer group, Ghana” through the hospital administration to assist the families in the cost of the treatment. Paediatric oncology nurses help to ensure continuity of care (Morrissey et al., 2019). In contrast to this assertion, Challinor at al. (2020) mentioned that although continuity and coordination of care seem straightforward, this process is often complicated by poor communication of goals and the imposition of personal beliefs maintained by members of the medical team (Challinor et al., 2020). Conversation between the patients, patients’ families and the healthcare team often does not occur promptly to have an impact on outcomes and improve the quality of life of children living with cancer (Jestico & Finlay, 2017; Morrissey et al., 2019). In this current study, the nurses explained that since they stay a longer time with patients, they were able to update the other cancer care, team members regularly. According to Sinclair et al. (2020), when paediatric care is well‐coordinated, it helps in preventing wastage of resources in the hospital, reduces the cost of hospitalization due to early discharge and helps improve clients and family's satisfaction with the care given by the oncology team (Sinclair et al., 2020). Oncology nurses interact with patients to ensure that they do not default in the cycle of chemotherapy treatment (Clark‐Snow et al., 2018).

This study explored nurses stories and has revealed the complexity of the phenomenon of caring for hospitalized children with cancer in the Ghanaian context. The Information contributed from nurses caring for oncological patients as a career specialization in this study can help in the identification of special needs for nurses and consequently ways appropriate support, encouragement and stability could be provided for the nurses. Increased awareness of this phenomenon for the nurses who care for these children is vital to ensure quality and holistic care that is meaningful and satisfying by the nurses for the patients.

The Hermeneutic phenomenology described by Hans Georg Gadamer and Diekelmann et al. approach involves the reader's interpretation of this work and the creation of their interpretations of the experience (Ramsbotham, 2019). Thus, it is acknowledged that in reading this work there is a possibility that it may help to clarify the experiences of caring for oncology patients in ways leading to insights about how nurses care for children with cancer in the Ghanaian context.

The findings endorse those from other small qualitative studies that highlight the meaning that nurses give to caring for hospitalized children with cancer and demonstrate the need for further research to generate more knowledge and understanding of this aspect of nurses’ work.

### 4.1 Strengths of the study

The ultimate purpose of this study was to gain an understanding of the lived experience of nurses caring for children hospitalised with cancer. This study has touched on how nurses feel, behave and how they cope with the struggles of life and even the death of their patients. The research gap that exists here in Tamale, Ghana on the absence of qualitative studies addressing the experience of nurses caring for children diagnosed with cancer has been filled through this research. This study is vital to ensure quality and holistic care that is meaningful and satisfying by the nurses for the patients. This study is essential for nurses and the science of nursing here in Ghana and around the globe on the importance of social support, strength and care to this delicate population of children. The results obtained from this study can be a motivation and guidance for the creation of the courses about how to care for children with cancer for nursing students.

### 4.2 Limitations of the study

The limitation of this research is that it is an interpretive analysis of the experience of a small group of nurses in a single hospital in Tamale, Northern part of Ghana, caring for children with cancer. The small sample size, type of qualitative study and sample selection process preclude generalization to all nurses working in the paediatric oncology unit. However, this is expected because phenomenological research aims to reveal the phenomena and understand the meaning of an experience, rather than generalizing or predicting the results of phenomena. Nevertheless, the stories reflected in this study are true descriptions of the phenomenon of caring among this group of nurses.

## 5 CONCLUSION OF THE STUDY

This study explored fourteen nurses' lived experiences of caring for hospitalized children with cancer and their families. The inclusion of the nurses' spoken words liberated meanings and experiences of caring for the children and their families, permitting insight into the intricacies of nurses caring roles as seen through the nurses' eyes. Understandings were discussed within the themes identified which were “Striving to reduce suffering,” including three relational subthemes: “knowing the children,” “rendering a hopeful fight” and “ensuring continuity and coordination of care.” This study showed some paths that have been formed and could undoubtedly contribute to reaffirm and relate with previous studies concerning the same theme, as well as to bring new knowledge to light. These shared practices and meanings sustain nurses as they cared for children with cancer daily. Thus, by striving to reduce suffering, paediatric oncology nurses created a caring atmosphere for these children with cancer and their families. Increased awareness of this phenomenon for the nurses who care for these children is vital to ensure quality and holistic care that is meaningful and satisfying by the nurses for the patients. Therefore, “striving to reduce suffering” should be understood by the paediatric oncology nurses as the meaning of caring for children with cancer.

## 6 RECOMMENDATIONS

Paediatric oncology nursing is a speciality that is unique that other nurses typically have little knowledge about it unless they are directly involved in caring for children with cancer. Consequently, the following recommendations were made:

- Nursing educators should focus on increased awareness of the caring roles of the paediatric oncology nurses in undergraduate and graduate nursing programmes.
- Another essential concern is for policymakers to formulate policies geared at paediatric cancer care through the study findings.
- Continued advancement of research exploration could be done to learn about the daily lived experiences of nurses who care for children with other chronic diseases in another context.
- A future research study in paediatric nursing oncology care could address nurses: personality traits, coping mechanisms, social, emotional and physical support based in Paediatric cancer care.

## Data Availability

The analysed transcripts data set used for this study will be made available from the corresponding author upon reasonable request.
